# Using a Systems Pharmacology Approach to Study the Effect of Statins on the Early Stage of Atherosclerosis in Humans

**DOI:** 10.1002/psp4.7

**Published:** 2014-12-30

**Authors:** C Pichardo–Almarza, L Metcalf, A Finkelstein, V Diaz-Zuccarini

**Affiliations:** 1 Department of Mechanical Engineering, University College LondonLondon, WC1E 7JE, UK; 2 Xenologiq LtdCanterbury, UK; 3 Department of Computer Science, University College LondonLondon, UK.

## Abstract

More than 100,000 people have participated in controlled trials of statins (lowering cholesterol drugs) since the introduction of lovastatin in the 1980s. Meta-analyses of this data have shown that statins have a beneficial effect on treated groups compared to control groups, reducing cardiovascular risk. Inhibiting the HMG-CoA reductase in the liver, statins can reduce cholesterol levels, thus reducing LDL levels in circulation. Published data from intravascular ultrasound studies (IVUS) was used in this work to develop and validate a unique integrative system model; this consisted of analyzing control groups from two randomized controlled statins trials (24/97 subjects respectively), one treated group (40 subjects, simvastatin trial), and 27 male subjects (simvastatin, pharmacokinetic study). The model allows to simulate the pharmacokinetics of statins and its effect on the dynamics of lipoproteins (e.g., LDL) and the inflammatory pathway while simultaneously exploring the effect of flow-related variables (e.g., wall shear stress) on atherosclerosis progression.

Absorption and metabolism of statins in humans have been analyzed using conventional pharmacokinetic and pharmacodynamic (PKPD) modeling approaches.^1^ The comprehensive, systematic and quantitative analysis of atherosclerosis formation is becoming essential now that patients are consistently treated with statins; particularly in the light of controversies over their benefit/detriment ratio (http://www.medscape.com/viewarticle/825246). Existing models can be improved by adding new mechanisms, and by providing a better description of the physiology and the PKPD properties of the drug. A systems pharmacology approach, aiming to provide a better model combining PKPD and systems biology, allows us to develop models containing more information about the dynamic interaction between a drug and a biological system so as to understand the behavior of the system as a whole, as opposed to the behavior of its individual components.^2^ As highlighted by Van der Graaf and Benson,^2^ systems pharmacology is expected to have an impact across all stages of drug research and development, ranging from very early discovery programs to large-scale Phase 3/4 patient studies. Our aim is to use the model proposed in this work to demonstrate how a “systems approach” can be used to develop a new model of atherosclerosis where mechanisms from different physical domains and biological scales can be combined with the PKPD properties of statins (i.e., simvastatin), with the objective to show plaque growth in a synthetic population using this approach and to compare this with well-known studies, for validation purposes.

## Mathematical models of atherosclerosis

Mathematical models of atherosclerosis pose a particular challenge in that they require the description of multiple physical (mechanical, biochemical, etc.), time-varying processes and mechanisms at multiple scales, i.e., from the physiological (i.e., blood hemodynamics) to nanoscale mechanisms in the arterial wall.^3^ One hypothesis, supported in the literature, is that mechanical factors such as wall shear stress, pulsatile blood flow, and vessel curvature are a trigger for the disease. This has been described in several models.^4^–^6^ In particular, wall shear stress (WSS) has been identified as one of the main hemodynamic factors determining endothelial dysfunction.^7^ Turbulence and flow detachment can be associated with the location of atherosclerotic plaque. The plaque specially affects “predisposed” areas of the blood vessel such as bifurcations or zones with flow recirculation, where the shear stresses are low. WSS lower than 0.4 Pa (approx.) stimulates an atherogenic phenotype and eases atherosclerotic lesion initiation.^7^ Conversely, literature also suggests an association between high shear stress values (i.e., greater than 1.5 Pa), endothelial quiescence, and atheroprotective gene expression.^7^

One key assumption in the modeling literature is that the main cause of plaque development is the abnormal enlargement of the intima by the infiltration and accumulation within the arterial wall of macromolecules such as lipoproteins, i.e., low density lipoprotein (LDL).^8^ Therefore, zones with high LDL values (which are zones likely to have pro-atherogenic WSS values) are considered to contribute to the development of the atherosclerotic lesion and intimal thickening. Additional mechanisms related to the recruitment of monocytes from the circulation (in response to inflammatory mediators) and their differentiation to macrophages to produce foam cells^9^ are also considered to model the formation of the fatty streaks.

The transport of macromolecules through the endothelium has been modeled previously considering two main pathways: transport through vesicular transcytosis (regulated by receptors on the endothelial cells)^10^ and transport through leaky and normal junctions, most of which are located at the sites of dying or replicating cells.^11^

A number of computational models have been proposed in the literature; for example, Cobbold^12^ and Gessaghi *et al*.^13^ modeled the oxidation process of LDL considering the action of different vitamins such as vitamin E in the LDL oxidation process. Di Tomaso *et al*.^14^ used a similar approach to model LDL oxidation coupled with a fluid dynamics model of the artery. Zohdi *et al*.^15^ considered the endothelial monocyte adhesion controlled by blood flow conditions and its influence on the inflammation process. Fok^16^ modeled the intimal thickening considering the release of cytokines and the migration of smooth muscle cells. Siogkas *et al*.^17^ used a similar approach including the dynamics of oxidized LDL, macrophages, and cytokines.

A similar model from Calvez *et al*.^18^ included the formation of foam cells. Ougrinovskaia *et al*.^19^ explored the uptake of cholesterol by different scavenger receptors of macrophages during early stage atherosclerosis. Bulelzai and Dubbeldam^20^ developed a simplified model for the dynamics of the main components of the atherosclerotic plaque: macrophages, monocytes, foam cell, and oxidized LDL. Chung and Vafai^21^ described the atheroma based on the LDL transport and how this was affected by fluid–structure interaction and pulsation. More recently Cilla *et al*.^22^ compiled and integrated several of the previous approaches in order to build a new model of atherosclerosis, including the species playing a main role in early development of the plaque, i.e., LDL, oxidized LDL, monocytes, macrophages, foam cells, smooth muscle cells, cytokines, and collagen.

The model presented in this work uses some of the assumptions from prior models, as explained below in the Methods section.

## Statins

Statins (e.g., Simvastatin) are a HMG-CoA (3-hydroxy-3-methylglutaryl-coenzyme A) reductase inhibitor effective in the treatment of hypercholesterolemia and hypertriglyceridaemia.^23^ They have been widely prescribed to reduce the risk of cardiovascular morbidity and mortality^24^ and thromboembolic stroke in high-risk patients.^25^,^26^ After oral administration, simvastatin is rapidly absorbed (max of 1–2 h)^27^,^28^ and eliminated. Once administered, simvastatin rapidly undergoes reversible nonenzymatic or carboxylesterase-mediated conversion to its active metabolite, simvastatin acid, in the liver, intestinal mucosa, and plasma.^23^,^29^ Simvastatin acid prevents HMG-CoA reductase from converting HMG-CoA to mevalonate, which is a rate-limiting step in cholesterol biosynthesis.^30^ Inhibition of the HMG-CoA reductase in the liver results in the reduction in cholesterol synthesis. Moreover, the up-regulation of LDL receptors located on the cell membranes of the liver and extrahepatic tissues thereby also contribute to the reduction in plasma LDL concentrations.^31^

Given the beneficial effects of statins in humans, the aim of this work is the development of a new integrative system model to yield a better understanding of the dynamics of the inflammatory mechanisms related to the early stages of atherosclerosis. Our model provides an account of the behavior of lipoprotein levels (e.g., LDL), the mechanical and flow properties inside the arterial lumen (e.g., wall shear stress), and the mechanisms of the effect of cholesterol lowering drugs (e.g., statins) on the progression of the atherosclerotic plaque.

## RESULTS

**Figure 1:**
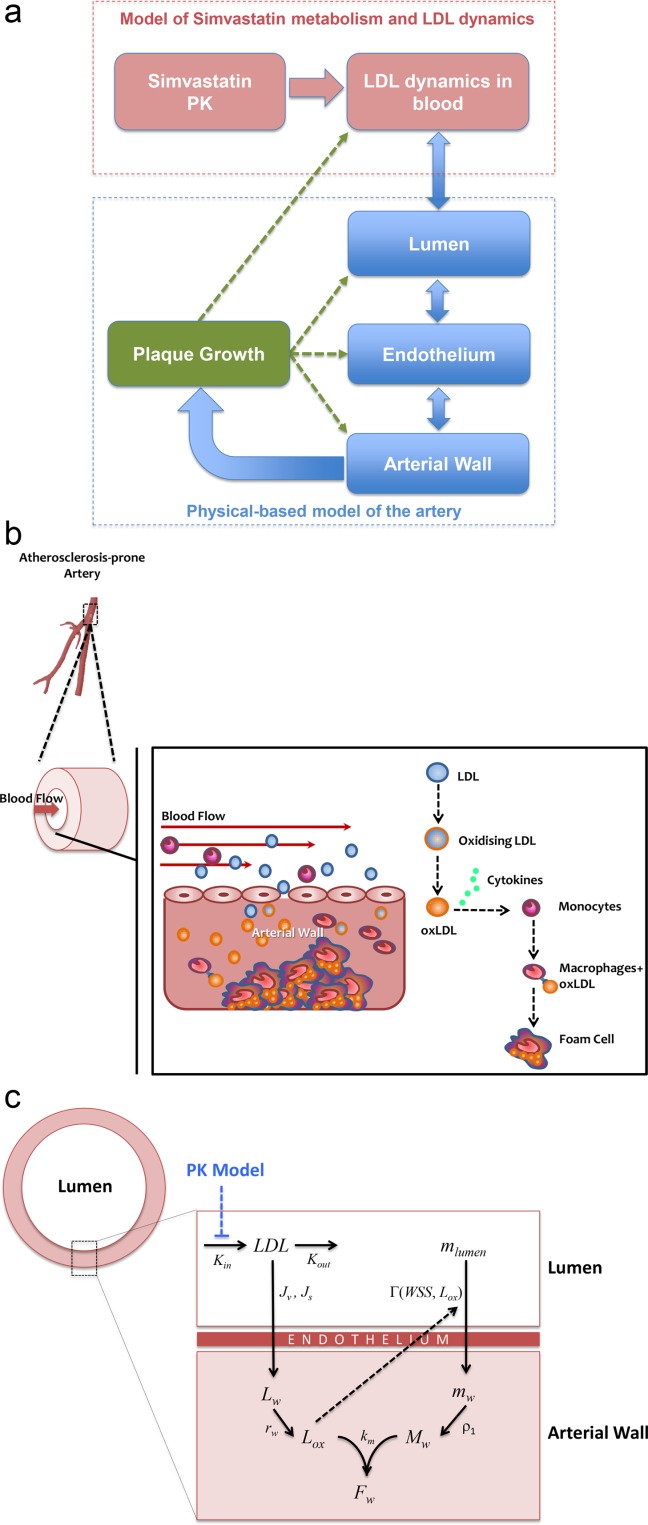
(a) Simplified diagram of the integrative systems model proposed. (b) Fatty streak formation: Main mechanisms considered in the atherosclerosis model. (c) Simplified diagram of the atherosclerosis model showing variables and kinetic rate constants.

The integrative model used in this work is the product of combining several approaches and temporal data related to the progression of the atherosclerotic lesion and the effect of statins inhibiting the development of the plaque. The final outcome shows the dynamic behavior of the plaque size, which has an effect on all the components of this model, mainly because species concentrations in the lumen and the arterial wall (and the WSS) are affected by changes in the arterial wall (see Figure 1a). Four main aspects were considered and combined to describe all these mechanisms and interactions: (1) blood flow and mechanical properties of the artery; (2) transport of macromolecules and immune cells through the endothelium; (3) administration, metabolism, and pharmacological effect of statins; (4) analysis and comparison of clinical trials.

**Figure 2:**
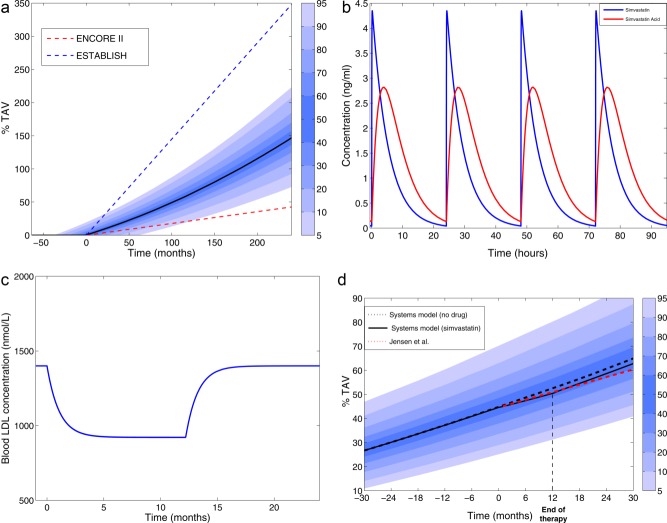
(a) Comparison of simulation results (%TAV - 10 years of plaque growth) with growth trends reported in literature for control groups. (b) PK of simvastatin and simvastatin acid using the pharmacokinetics/pharmacodynamics (PKPD) model proposed. (c) LDL dynamic response to a daily dose of 40 mg of simvastatin during 12 months. (d) Comparison of simulation results for the plaque growth (%TAV) with the Jensen *et al*.^32^ study. In panel (a), time = 0 months is the time when the plaque starts to grow. In panels (b–d), time = 0 months is the time when the therapy starts.

**Figure** 2**a** illustrates the behavior of the model when there is no drug present in the system. The black line is the median of %TAV for the synthetic population. The dotted red and blue lines show a projection of the trends using the growth rates reported in ESTABLISH^33^ and ENCORE II^34^ studies (see Supplementary Materials S1, available online). Using the parameter values proposed in this work, the 5^th^ and the 95^th^ percentile of the simulated values are between the boundary values of growth imposed by the data estimated from these clinical trials. It is possible to increase the variability of this variable (%TAV) expanding the variation range of the parameters and/or using a smaller synthetic population (< 1,000 individuals).

The simulated response curve of simvastatin and simvastatin acid for the first 96 h (four administrations) are shown in **Figure** 2**b**. According to the measurements of Kim *et al*.,^1^ the half-life of simvastatin is relatively short (2–5 h), in fact plasma concentration of simvastatin is eliminated in 2 d after single administration^1^ (not shown in **Figure** 2**b** due to the daily dose regimen).

The simulated LDL concentrations, however, continued to decrease when a multiple dose is applied (**Figure** 2**c**). Similar results have been described in the label of ZOCOR (http://www.merck.com/product/usa/pi_circulars/z/zocor/zocor_pi.pdf) and another simvastatin study reported by Law *et al*.^26^

**Figure** 2**d** illustrates the comparison of the simulation results of the proposed integrative model with the growing trends obtained from the study by Jensen *et al*.^32^ The decrease of LDL in the circulation due to the effect of simvastatin produced a decrease on the amount of LDL entering the wall and the production of foam cells leading to a decrease on the plaque rate of growth, as shown in **Figure** 2**d**.

Furthermore, the model shows the effects of blood flow on the initiation and development of atherosclerotic plaque. Changes in lumen diameter owing to plaque growth will influence the pattern of blood flow, and therefore WSS,^35^ with the endothelial cells responding to signals induced by WSS.^35^,^36^ Therefore, the low shear stress region of the plaque will remain atheroprone.

## METHODS

### Mathematical modeling of atherosclerosis

The mathematical model presented here is based upon the hypothesis that the atherosclerosis process begins with the entry of LDL into the intima of the vessel,^39^ where it oxidizes, starting the inflammatory process: monocytes first adhere to the endothelium, and then penetrate into the intima, where they differentiate into macrophages (see **Figure** 1**b**).

The secretion of pro-inflammatory cytokines (due to a chronic inflammatory response) promotes the recruitment of monocytes from the circulation, which differentiate to macrophages in the arterial wall. Macrophages uptake of oxidized LDL (ox-LDL) leads to the formation of foam cells, which in turn may be removed by the immune system.

This model (described in words above) is conceptually divided in two main parts: a “biochemical” model describing the LDL oxidation and the immune response (e.g., effect of pro and anti-inflammatory cytokines); and a “mechanical” model describing the plaque growth and the effect of blood flow conditions on the evolution of the plaque. The atherosclerotic lesion, comprising fibrous cap and lipid deposit, changes the internal geometry of the vessel (lumen) and interacts with the blood flow. This interaction may lead to a thrombus, or to the degradation and rupture of the plaque.^40^ In this model, low shear stress is associated with plaque location, given some evidence suggesting that shear stress may act through endothelial gene expression or by a process of localized inflammation.^41^ A simplified diagram of the processes described by this mathematical model is shown in **Figure** 1**b**.

#### Modeling the hemodynamics, endothelial behavior, and WSS

Capturing the dynamics of the blood flow is fundamental as atherosclerotic-prone areas are associated with regions of low shear stress due to their impact on endothelium behavior as previously mentioned. The hemodynamics are calculated using the continuity (1) and Navier-Stokes equations 2.

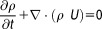
1

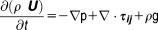
2where *p* is the pressure, τ is the shear stress tensor, ρ is the density, and *U* is the flow velocity.

Local flow conditions near the wall can significantly enhance or impede the transport of molecules from the lumen to the arterial wall and are typically characterized by the WSS. For fully developed, laminar flow through a rigid tube and using Poiseuille law, τ can be calculated as a function of the volumetric flow rate (*Q*), the viscosity of the blood (μ), and the vessel radius (*r*)^42^ as follows:

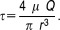
3

#### Membrane-transport model

The LDL flux across the endothelium was modeled using Kedem Katchalsky's equations, as below.

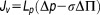
4

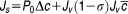
5where *J_v_* is the transmural velocity and *J_s_* is the solute flux through the endothelium, *Lp* is the hydraulic conductivity, Δ*p* and ΔΠ are the pressure difference and the osmotic pressure difference across the endothelial membranes, respectively, *P_0_* is the diffusive endothelial permeability, Δ*c* is the solute concentration,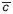 is the mean endothelial concentration, σ is the reflection coefficient. The osmotic pressure difference was neglected to decouple the fluid dynamics from solute dynamics.^43^,^44^ It is assumed that the hydraulic conductivity depends on the shear stress (3) at the wall (WSS), using the following equation^45^:

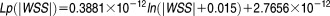
6where |*WSS*| is the absolute value of WSS. This relationship between WSS and *Lp* shows that the endothelium permeability to blood macromolecules increases when subject to higher WSS. The hemodynamic stimulus on endothelial cells is mediated by mechanoreceptors linked to critical signaling pathways (e.g., NO-dependent mechanisms) and involves the conversion of the biomechanical stimuli to biochemical responses. In this context, the above relationship incorporates into the simulation the effect of WSS on cellular processes that result in the alteration of the pathways governing the transendothelial transport of water and solutes (e.g., intercellular junctions).^46^,^47^

An additional relationship between the diffusive permeability and LDL concentration was proposed by Sakellarios *et al*.^45^ on the basis of experimental data reported by Guretzki *et al*.^48^ (Eq. 7):

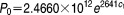
7where *c*_1_ is the local LDL concentration on the luminal side of the endothelium. Using this equation, the LDL endothelial penetration depends on WSS as well as the magnitude of the lumen concentration.

#### Atherogenesis as a transport process inside the arterial wall

The intima, media, internal elastic lamina, and external elastic lamina were considered as forming one single layer. The results shown in this article are from a simplified version of the model where the arterial wall is considered a well-stirred volume, given that results of the spatial distribution of the different species in the wall (e.g., LDL, foam cells, etc.) are out of the scope of this work. In general, atherosclerosis models use a convection-diffusion-reaction equation to describe the transport of LDL (*L_w_*) from the arterial lumen to the arterial wall (see for example the model from Di Tomaso *et al*.^14^):

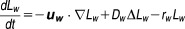
8where ***u_w_*** is the transmural velocity and *D_w_* is the diffusivity of LDL in the wall. The last term of Eq. 8 represents the oxidation of LDL, where *r_w_* is the reaction rate constant. The oxidized LDL (*L_ox_*) is then expressed as:

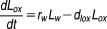
9

The transport of monocytes in the arterial wall (*m_w_*) is controlled by *L_ox_* and the concentration of monocytes in the lumen (*m_lumen_*) (assumed constant for this first modeling approach). This is calculated using a similar relationship to the one proposed by Bulelzai and Dubbeldam^20^ and Cilla *et al*.^22^:

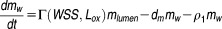
10

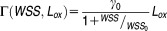
11where ρ_1_ is the rate constant for the differentiation of monocytes into macrophages. The value of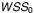 designates the wall shear stress at which the growth rate of the monocyte concentration due to the signaling response by the endothelium is reduced with a factor of two compared to the zero wall shear rate response by the endothelium. The factor γ_0_ is a constant determining the rate at which monocytes enter the intima for low wall shear stress and low *L_ox_*.

Finally, the dynamic equations of the macrophages (*M_w_*) and the formation of foam cells (*F_w_*) due to the macrophage uptake of oxidized LDL (*L_ox_*) inside the arterial wall are:

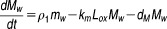
12

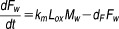
13where *d_lox_*, *d_m_*, *d_M_*, and *d_F_* are rate constants for oxidized LDL, monocytes, macrophages, and foam cell diffusion out of the plaque respectively.

**Figure 3:**
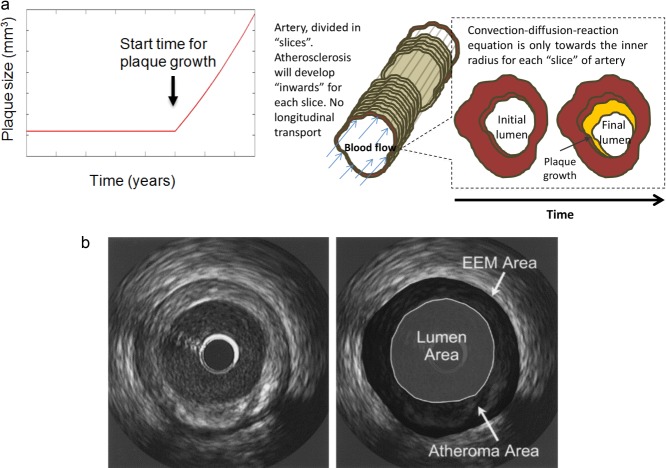
(a) “Growth” as implemented in the model. The arterial lumen (in red) would be remodeled as a consequence of plaque formation and development in time (in yellow). (b) Method for analysis of atheroma area. The left panel shows a representative intravascular ultrasound cross section. The right panel illustrates the boundaries planimetered for the external elastic membrane (EEM) and lumen and how the atheroma area is calculated. Image published by Nicholls *et al*.^49^ Copyright Elsevier (2006).

To simulate the formation of the initial stages of the plaque, it was assumed that a narrowing of the lumen would occur if the volume covered by all the cells considered is larger than the initial arterial volume (**Figure** 3**a**). Please note that foam cells are key for the remodeling of the arterial wall.

The volume of the plaque (i.e., intima plus media layers) is then calculated as the sum of the volumes occupied by the different species (i.e., foam cells, macrophages, monocytes, LDL) inside the wall.

### PKPD model of statins

The PKPD model used in this work is based on the model published by Kim *et al*.^1^ The model used well stirred compartments (assuming that the mixing rates are shorter than the reaction rates) and was developed using data collected from 27 healthy male volunteers with a daily dose of 40 mg of simvastatin given for 14 d. Simvastatin lactone (parent drug) and simvastatin acid (metabolite) plasma concentrations were measured at 0, 0.5, 1, 1.5, 2, 3, 3.5, 4, 5, 6, 8, 10, 12, and 24 h after the dose on days 1, 7, and 14. LDL concentrations were measured daily after an overnight fast.

**Figure 4:**
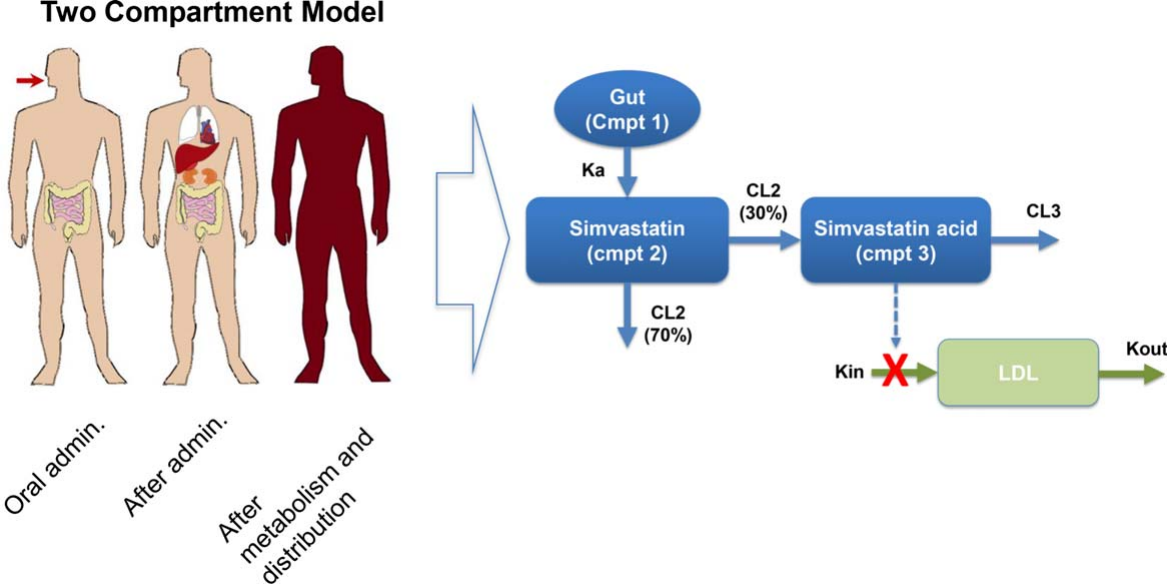
Diagram of the pharmacokinetics/pharmacodynamics (PKPD) model used in this work and proposed by Kim *et al*.^1^

The PK model is a parent/metabolite model with first-order absorption and elimination as this configuration provided the best fit for the pharmacokinetic data. The PD model is based on the effect of simvastatin on LDL concentration using an inhibitory turnover model with the metabolite, simvastatin acid, as the driver (**Figure** 4). **Table** 1 summarizes the population parameter estimates for this model and published by Kim *et al*.^1^

**Table 1:** Systems pharmacology model: parameter values

Symbol	Quantity	Value
Model of atherosclerosis		
*k_m_* (m^3^ cells^−1^ s^−1^)	Foam cell formation constant^22^	9.25 × 10^−24^
*d_m_* (s^−1^)	Diffusion of monocytes out of the plaque^20^	5.75 × 10^−6^
*d_lox_* (s^−1^)	Diffusion of LDL out of the plaque^20^	2.4 × 10^−5^
*d_M_* (s^−1^)	Diffusion of macrophages out of the plaque^a^	5.75 × 10^−6^
*d_F_* (s^−1^)	Diffusion of foam cells out of the plaque^a^	5.75 × 10^−6^
*r_w_* (s^−1^)	LDL oxidation rate constant^14^	3 ×10^−4^
*µ*(Pa s)	Blood viscosity^20^	0.004
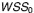 (Pa)	WSS threshold for monocyte wall transport^20^	1
ρ_1_ (s^−1^)	Rate of differentiation of monocytes into macrophages^20^	1.15 × 10^−6^
*r*_0_ (dm)	Initial lumen radius	0.03
*γ*_0_ (m^3^ nmol^−1^ day^−1^)	Rate constant in Eq. 11,^22^	5.5 × 10^−13^
Δ*p* (Pa)	Endothelial pressure difference^22^	2400
*Q* (l/s)	Blood flow	0.0075
*m_lumen_* (cells/l)	Concentration of monocytes in blood^22^	5.5 × 10^8^
σ	Endothelial reflection coefficient^45^	0.997
PKPD Model		
CL2(L/hr)	Clearance compartment 2	1740
V2(L)	Volume compartment 2	8980
CL3(L/hr)	Clearance compartment 3	383
V3 (L)	Volume compartment 3	1190
Ka (1/hr)	Absorption rate constant	2.76
K_in_ (nmol/L/hr)	Production rate of LDL	29.52
Emax	Max. effect due to the drug	0.489
EC_50_ (ng/ml)	Blood concentration at half max. effect	0.0868
LDL_baseline_ (nmol/L)	Baseline LDL	1400
K_out_	Elimination rate constant for LDL	Kin/LDL_baseline_

a Value proposed for this model.

The two-compartment model shown in **Figure** 4 tries to reproduce the temporal data related to the absorption and metabolism of the drug (in this case, simvastatin), its pharmacological effect on the production of cholesterol by the liver and the availability of LDL in circulation. The mathematical equations to describe these mechanisms are ordinary differential equations related to a standard two-compartment PKPD model.^1^

### Clinical trials and assessment of the atheroma burden

IVUS studies were selected to evaluate the effect of statins in the progression of the atheroma size. IVUS is a medical imaging methodology using a catheter with a miniaturized ultrasound probe attached that provides high-resolution information about the arterial lumen and wall. **Figure** 3**b** shows how IVUS are used to estimate the volume of the plaque.

The results of the control groups reported in the ESTABLISH^33^ and ENCORE II^34^ trials were used to compare with the results of the integrative model when there is no drug in the system.

The results of plaque growth under the effect of simvastatin reported by Jensen *et al*.^32^ were used to compare the results of the integrative model in the presence of the drug. A brief summary of the results from these trials is shown in **Table** 2.

**Table 2:** Intravascular ultrasound progression/regression studies

Study	Type	Year	Treatment	*n* (subjects)	Follow-up (months)	% Change of TAV
ESTABLISH^33^	RCT	2004	Atorvastatin	24	6	13.1%
			Control	24		8.7%
ENCORE II^34^	RCT	2009	Nifedipine	97	18–24	5.0%
			Placebo	96		3.2%
Jensen et al.^32^	Non-RCT	2004	Simvastatin	40	12	6.3%

RCT, randomized controlled trial.

The atheroma or plaque area in cross-sectional IVUS images was calculated by subtracting the lumen area from the external elastic membrane area (see **Figure** 3**b**). Hence, an IVUS-defined atheroma area is a combination of plaque plus media area. The atheroma area is calculated in each frame (cross-sectional image), and total atheroma volume (TAV) (Eq. 14) can be calculated based on the pullback speed during imaging acquisition. Then, the change of the atheroma volume can be reported as a percentage of the initial volume of the external elastic membrane occupied by atheroma, called in our case %TAV (Eq. 15). (The reader is referred to the work by Garcia-Garcia *et al*.^50^ for more details about these methods.)

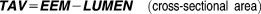
14

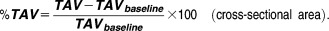
15

These equations give an estimation of the changes of %TAV that can be compared with the simulation results from the model proposed.

### Simulations: synthetic population

As an illustrative example for this article a synthetic population was built in order to evaluate how the simulations behave with respect to the variability of some parameters between individuals. Simulations were generated with Matlab (http://www.mathworks.com) using the ode15s solver (Supplementary Materials S2 includes more details about the computational implementation of this model).

The population is composed of 1,000 subjects. Based on the data available, a minimal set of parameters was varied in order to produce individual variations. Three model parameters were selected considering they were log-normal distributed across the population. They were the LDL concentration in blood, the blood viscosity, and the radius of the artery, parameters that are expected to show some variability in clinical studies, however, additional parameters (i.e., kinetic rate constants) might be included in future simulations in order to include additional intervariability between patients, although in that case additional data (i.e., more complex measurements and experiments related to the kinetic rates) might be needed.

**Table** 3 summarizes the statistical details of these parameters used for the simulations.

**Table 3:** Parameter values for the synthetic population

LDL levels in blood:
Mean = 1402 nmol/l
Median = 1396 nmol/l
Standard deviation = 141
5^th^ and 95^th^ percentile = (1190 – 1644)
Blood viscosity:
Mean = 0.004 Pa s
Median = 0.004 Pa s
Standard deviation= 8e-5
5^th^ and 95^th^ percentile = (0.0039 – 0.0041)
Lumen radius:
Mean = 0.03 dm
Median = 0.03 dm
Standard deviation= 0.0015
5^th^ and 95^th^ percentile = (0.0277 – 0.0325)

## DISCUSSION

Results of the coupling between the PKPD model and the more detailed model of the artery shows the effect of statins on several mechanisms related to plaque growth, beyond the effect that statins have shown lowering LDL values in circulation. A comparison with the results reported by Jensen *et al*.^32^ (**Figure** 2**d**) has shown how this type of systems model can be calibrated in order to reproduce the dynamic response of the plaque growth for a given population treated with simvastatin.

Simulation results allow us to conclude that new integrative models can be developed combining information and data from different sources in order to achieve a holistic understanding of the process of atherosclerosis and the effect of statins. The model presented in this paper is modular, meaning that specific parts of the model (modules) were developed to describe specific mechanisms, i.e., a module to describe blood flow, another module to describe LDL dynamics and oxidation, another module to describe immune response, and another module to describe statin administration, metabolism, and pharmacological effect. The main advantage of this approach is that each individual module can be improved (for example, by adding more mechanisms based on new evidence or data) or simplified being the ultimate goal the validation of all the biological/physiological scales considered in this type of model. More specifically, future changes in the PKPD module to improve the whole model might consider:

1. Modeling the effect of circadian rhythms on the LDL levels as proposed by Wright *et al*.^37^
2. The inclusion of the pleiotropic effect of statins, using a similar approach to that proposed by Rose *et al*.^38^ (modifying the PK model proposed here)
3. Studying the effect of statins on the upregulation of hepatic LDL receptors

Here, an analysis based on the effects of simvastatin was made, however other cholesterol-lowering drugs could also be considered using the same approach just by changing the PKPD properties.

The absorption and metabolism of statins in humans have been analyzed before using conventional pharmacokinetics and pharmacodynamics (PKPD) modeling approaches.^1^ Our work has shown that these models can be improved adding new mechanisms and providing a better description of the physiology and the PKPD properties of the drug. A systems pharmacology approach was applied, combining PKPD and physiological modeling based on mass transport and fluid dynamics, giving more information about the dynamic interaction of different elements of the atherosclerotic plaque and the drug. The model showed how a systems pharmacology approach can be used to develop a new model of atherosclerosis where mechanisms from different physical domains and biological scales can be combined with the PKPD properties of statins (i.e., simvastatin).

It is important to mention that the upregulation of LDL receptors in hepatocytes is not included in this model. The model focuses on the plaque formation process, *inside the arterial wall*, with LDL as the main trigger for the atherosclerotic process to occur (cause-effect relationship). In this case, the effect of statins as a way to decrease LDL levels is explored.

## Study Highlights

WHAT IS THE CURRENT KNOWLEDGE ON THE TOPIC?

✓ Prior research has studied statins using standard PKPD modeling approaches. There are currently no models in the literature coupling the PK properties of statins and their effect on the atherosclerosis inflammatory mechanisms and mechanical properties in arteries.

WHAT QUESTION DID THIS STUDY ADDRESS?

✓ We seek to improve the collective understanding of the effect of cholesterol-lowering drugs in the development of plaque by using a novel modeling approach able to capture mechanistically, the relationships between the drug and a physiological representation of the artery.

WHAT THIS STUDY ADDS TO OUR KNOWLEDGE

✓ Data on lipid metabolism and atherosclerosis is increasingly available and further experimental and clinical studies are anticipated. The systems model proposed here shows how clinical trials data can be combined with mechanistic models of atherosclerosis.

HOW THIS MIGHT CHANGE CLINICAL PHARMACOLOGY AND THERAPEUTICS

✓ This kind of integrative approach permits the study of the effect of statins beyond lowering cholesterol levels in the circulation. Computational simulations can test different pharmacological or physiological hypotheses on atherosclerosis progression, with potential benefits to health outcomes.

## Supporting Information

Supplementary information accompanies this paper on the CPT: Pharmacometrics & Systems Pharmacology website (http://www.wileyonlinelibrary.com/psp4)
