# Supplementary material for: Using a Systems Pharmacology Approach to Study the Effect of Statins on the Early Stage of Atherosclerosis in Humans
Source: CPT Pharmacometrics Syst Pharmacol. 2014 Dec 30;4(1):e00007. doi: 10.1002/psp4.7 (PMC4337252; doi:10.1002/psp4.7)
Supplement: Supplementary file 1 [file psp40004-e00007-sd1.docx]

**Appendix 1. Description of ESTABLISH**^1^ **and ENCORE II**^2^

Baseline characteristics of patients

|  | ESTABLISH  Control | ENCORE II  Placebo |
| --- | --- | --- |
| n | 35 | 112 |
| Gender: males (%) | 30 (85.7) | 93 (83) |
| Age, years (±SD) | 62.5 (±11.2) | 57.4 (±8.8) |
| BMI, kg/m^2^ | 24.0 (±3.2) | 27.4(±3.4) |
| Smoking, n (%) | 24 (68.6) | 29 (24.7%) |

Blood parameters of patients at Baseline and Follow-Up

|  | ESTABLISH  Control | | ENCORE II  Placebo | |
| --- | --- | --- | --- | --- |
|  | Baseline | Follow-Up  (6 months) | Baseline | Follow-Up  (18-24 months) |
| Total cholesterol, mg/dL (±SD) | 190.7 (±42.8) | 190.9 (±29.7) | 199.9 (±11.3) | 187.1 (±41.3) |
| HDL-C,  mg/dL (±SD) | 44.3 (±11.2) | 47.4 (±11.2) | 37.6 (±11.3) | 40.8 (±11.3) |
| LDL-C,  mg/dL (±SD) | 123.9 (±35.3) | 119.4(±24.6) | 124.6 (±38) | 109.1 (±33.8) |
| Triglycerides,  mg/dL (±SD) | 112.3 (±54.5) | 120.4 (±49) | 162 (±95) |  |

**References**

1. Okazaki, S. *et al.* Early statin treatment in patients with acute coronary syndrome: demonstration of the beneficial effect on atherosclerotic lesions by serial volumetric intravascular ultrasound analysis during half a year after coronary event: the ESTABLISH Study. *Circulation* **110,** 1061–1068 (2004).

2. Lüscher, T. F. *et al.* A randomized placebo-controlled study on the effect of nifedipine on coronary endothelial function and plaque formation in patients with coronary artery disease: the ENCORE II study. *Eur. Heart J.* **30,** 1590–1597 (2009).
