# Supplementary material for: Using a Systems Pharmacology Approach to Study the Effect of Statins on the Early Stage of Atherosclerosis in Humans
Source: CPT Pharmacometrics Syst Pharmacol. 2014 Dec 30;4(1):e00007. doi: 10.1002/psp4.7 (PMC4337252; doi:10.1002/psp4.7)
Supplement: Supplementary file 2 [file psp40004-e00007-sd2.docx]

Pseudo code of the mathematical model of atherosclerosis proposed

%% Atherosclerosis Model %%

% Define locations of interest

compartment_of_interest = compartments defined by the modeller for mass balances

% Define ODEs:

% ODE for LDL

d(LDL@compartment_of_interest)/dt = Sum (pertinent_ReactionFlux(@compartment_of_interest))

% ODE for oxidised LDL

d(oxidised LDL)/dt = Sum (pertinent_ReactionFlux(@compartment_of_interest))

%ODE for monocytes

d(monocytes@compartment_of_interest)/dt = Sum (pertinent_ReactionFlux(@compartment_of_interest))

%ODE for macrophages

d(macrophages)/dt = Sum (pertinent_ReactionFlux(@compartment_of_interest))

%ODE for foam cells

d(foam_cells)/dt = Sum (pertinent_ReactionFlux(@compartment_of_interest))

% Fluxes:

% ReactionFlux1 = LDL endothelial transport

% ReactionFlux2 = LDL oxidation rate

% ReactionFlux3 = Foam Cell formation rate

% ReactionFlux4 = Macrophage formation rate

% ReactionFlux5 = Monocytes endothelial transport

% ReactionFlux6 = Degradation of foam cells

% ReactionFlux7 = Degradation of oxidised LDL

% ReactionFlux8 = Degradation of macrophages

% ReactionFlux9 = Degradation of LDL

% ReactionFlux10 = Degradation of monocytes

ReactionFlux1 = f1(LDL, wss, deltaP)

ReactionFlux2 = f2(LDL)

ReactionFlux3 = f3(oxLDL, Macrophages)

ReactionFlux4 = f4(Monocytes)

ReactionFlux5 = f5(oxLDL, Monocytes, wss)

ReactionFlux6 = f6(Foam_Cells)

ReactionFlux7 = f7(oxLDL)

ReactionFlux8 = f8(Macrophages)

ReactionFlux9 = f9(LDL)

ReactionFlux10 = f10(Monocytes)

% Additional algebraic equations:

% Volume of LDL particles

V_LDL_part = f11(LDL, oxLDL)

%Volume of cells

V_cells = f12(Foam_Cells, Monocytes, Macrophages)

%Total volume

Wall = f13(Initial_volume + remodelling)

%Calculate dynamic volume of the wall (initial and increments) (PATV = %TAV)

PTAV = f14(Wall, Initial Wall)

% Calculate Volume of the Lumen

Lumen = f15(Wall)

%Radius of the Lumen

Rlumen = f16(Lumen)

%Wall shear stress

wss = f17(Blood flow, blood viscosity, Rlumen)
